# Efficacy of pregabalin for the treatment of chronic pruritus of unknown origin, assessed based on electric current perception threshold

**DOI:** 10.1038/s41598-020-57629-z

**Published:** 2020-01-23

**Authors:** JaeIn Lee, DongHyek Jang, JooYoon Bae, HyeJung Jung, MiYoun Park, JiYoung Ahn

**Affiliations:** Department of Dermatology, National Medical Center, Seoul, Republic of Korea

**Keywords:** Skin diseases, Skin manifestations

## Abstract

Chronic pruritus of unknown origin (CPUO) is defined as itching lasting more than 6 weeks in the absence of discernible skin lesions. Pregabalin is used to treat patients with CPUO. In this study, we aimed to investigate differences in the perception threshold of itch sensation between patients with CPUO and healthy individuals and to evaluate the efficacy of pregabalin for CPUO. At baseline, week 2, and week 4 after treatment initiation, the visual analogue scale (VAS) score was measured to assess pruritus severity, and electric current perception threshold (CPT) was measured at 250 and 5 Hz using a NEUROMETER CPT/C stimulator. Twenty healthy individuals and 41 patients with CPUO were enrolled in this study. The patients with CPUO were categorised as those who responded to antihistamines (Antihistamine group), were not improved by antihistamines (Pregabalin group), and were not improved by antihistamines and pregabalin (Refractory group). The baseline CPT values were not significantly different between patients with CPUO and healthy control. Pruritus was improved in 7 of 10 patients in the Pregabalin group after treatment with pregabalin, showing decreased CPT at 5 Hz. The sensitive C-fibres presented a high threshold to detect itch sensation, and this sensitivity decreased in response to treatment with pregabalin.

## Introduction

Chronic pruritus is defined as itching that persists for more than 6 weeks^1^. Chronic pruritus of unknown origin (CPUO) refers to the condition where such itching occurs in the absence of known disease processes^2,3^. Given that patients with CPUO often fail to respond to topical treatments and oral antihistamines, gabapentin or pregabalin, which are used to treat neuropathic pain^4^, have been increasingly used because CPUO and neuropathic pain are associated with a similar involvement of the nervous system^5^.

Measurement of the electric current perception threshold (CPT) is based on the detection of the threshold of sensory nerves to electric stimulation^6^. NEUROMETER CPT⁄C (Neurotron Inc., Baltimore, MD, USA) measures the CPT at frequencies of 2,000, 250, and 5 Hz, which are the sensory thresholds of Aβ-, Aδ-, and C-fibres, respectively^6^, of which Aδ- and C-fibres are considered to transmit itching signals from the skin^7^.

To the best of our knowledge, no study has evaluated the CPT in patients with CPUO during pregabalin treatment and compared their baseline data with those of healthy people. Therefore, we attempted to confirm, using the visual analogue scale (VAS) and CPT, the effect of pregabalin on patients with CPUO who were unresponsive to topical steroids and oral antihistamines. We also investigated whether there are any differences in the CPT between patients with CPUO and healthy individuals.

## Methods

### Study participants

Eligible participants were adults with chronic pruritus that persisted for more than 6 weeks and healthy individuals as the Healthy control group. Patients with chronic pruritus had thorough history taking, physical examination, and laboratory tests, including tests for creatinine, aspartate aminotransaminase (AST), alanine aminotransferase (ALT), alkaline phosphatase, bilirubin, thyroid stimulating hormone (TSH), complete blood count, and glucose, and chest X-ray. To enrol the patients with CPUO, patients with chronic pruritus were excluded if they had one or more of the following: a history of diabetes mellitus, hepatobiliary disease, chronic renal failure, eczema, psoriasis, and other skin diseases with overt inflammation improved by topical steroids or oral antihistamines, abnormal laboratory results, and an initial VAS score of lower than 3 or a duration of pruritus of less than 6 weeks. Data were collected at the Department of Dermatology of the National Medical Center, Seoul, South Korea. The period of the study was from October 19, 2017, to October 18, 2018. The protocol of this study was reviewed and approved by the National Medical Center Institutional Review Board [H-1708-081-001]. All clinical investigations were conducted according to the principles expressed in the Declaration of Helsinki. Written informed consent was obtained from all participants.

### Study design and intervention

At the time of screening, the patient’s eligibility was assessed, and demographics and medical history were documented. A single dermatologist assigned the baseline VAS scores and obtained the CPT values. We assessed pruritus using the VAS, ranging from 0 (no pruritus) to 10 (extremely severe pruritus), at baseline, 2 weeks, and 4 weeks, based on the patient’s response to the question: “How much did you itch over the past 24 hours?” CPT was measured using NEUROMETER CPT/C, a neuroselective transcutaneous electrical stimulator that evokes electrical itch in a non-invasive fashion, at frequencies of 5 and 250 Hz on one forearm of the patients with CPUO and healthy controls. Specifically, the current was delivered to the skin using a pair of 1-cm diameter gold surface electrodes that were covered by a thin layer of electroconductive gel and separated by 1.7 cm with a clear mylar spreader.

**Figure 1:**
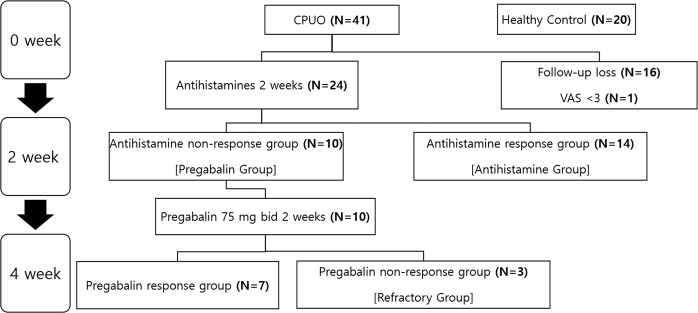
Diagram of participant recruitment and flow. CPUO, chronic pruritus of unknown origin.

Patients with CPUO were prescribed two types of second-generation antihistamines and instructed to avoid any other therapeutic agents for relieving pruritus, including topical corticosteroids. These patients were assessed 2 weeks after the commencement of treatment. At the 2-week follow-up visit, a single dermatologist assigned the VAS scores and measured the CPT on one forearm. Patients who reported a decrease of two or more points in the VAS score were assigned to the Antihistamine group. Patients who reported no change, a decrease of only one point, or an increase in the VAS score were assigned to the Pregabalin group and were additionally prescribed pregabalin at 150 mg/day for 2 weeks. At the subsequent follow-up visit, a single dermatologist assigned the VAS score, measured the CPT on one forearm, and recorded any adverse events. Patients who reported no change, a decrease of only one point, or an increase in the VAS score at 4 weeks were assigned to the Refractory group. We defined improvement in pruritus as a decrease in the VAS score of two or more points (Fig. 1).

### Statistical analysis

The baseline VAS scores and CPT values were compared among the Antihistamine, Pregabalin, Refractory, and Healthy control groups using analysis of variance, whereas changes in the CPT and VAS values were analysed using a paired *t*-test. The threshold of statistical significance was a two-sided p-value of 0.05. Statistical analysis was performed using R statistical package, version 3.5.0^8^.

## Results

### Participants’ characteristics

Of the 61 participants enrolled in this study, 20 were healthy control individuals, and the remaining 41 were patients with CPUO. All patients with CPUO did not have other comorbidities. Among the 41 patients with CPUO, 16 patients who were lost to follow-up after baseline evaluation were excluded from data analysis. One more patient was excluded based on the VAS score of lower than 3. The remaining 24 patients with CPUO completed the study and were subsequently included in the analysis (Fig. 1). The reported adverse events were somnolence (n = 2) and dizziness (n = 2). Most patients tolerated pregabalin well.

The demographics of the study participants are listed in Table 1. The Refractory group showed the highest average mean age of patients (69.7 years), followed by the Pregabalin (62.9 years), Antihistamine (62.0 years), and Healthy control (43.9 years) groups. These differences were significant (p = 0.0003).

**Table 1:** Demographics of the participants.

Characteristics	Antihistamine group (N = 14)	Pregabalin group (N = 10)	Refractory group (N = 3)	Healthy control group (N = 20)	p-value
Sex (Male), No. (%)	9 (64.3%)	7 (70.0%)	3 (100%)	8 (20.0%)	0.15
Age (years), [95% CI]	62.0 [58.0–66.0]	62.9 [55.1–70.7]	69.7 [39.6–99.7]	43.9 [35.2–52.5]	0.0003
Initial VAS [95% CI]	7.1 [5.9–8.3]	5.9 [3.9–7.9]	4.3 [−1.4 to 10.1]	NA	
Duration (months) [95% CI]	62.5 [8.1–117]	46.8 [−3.4 to 97.1]	16.2 [−26.7 to 59.1]	NA	

NA, Not applicable.

### Results obtained at 5 Hz

The mean baseline CPT values for different groups, measured at 5 Hz on one forearm, were as follows: the Antihistamine group: 28.0; the Pregabalin group: 26.4; the Refractory group: 23.6; and the Healthy control group: 22.1. The mean CPT values recorded at 5 Hz on one forearm of patients in the Antihistamine, Pregabalin, and Refractory groups at weeks 2 and 4 are shown in Table 2.

**Table 2:** Baseline, week 2, and week 4 CPT values at 5 Hz.

CPT 5 Hz (C-fibres)	Mean [SD] CPT values			
	Antihistamine group	Pregabalin group	Refractory group	Healthy control group
Baseline	28.0 [23.9]	26.4 [12.6]	23.6 [15.5]	22.1 [10.6]
Week 2	17.4 [12.1]	28.4 [16.7]	18.3 [6.5]	NA
Week 4	NA	16.3 [9.3]*	22.7 [6.4]	NA

CPT, current perception threshold. NA, Not applicable.

At baseline, the CPT values were compared among the Antihistamine, Pregabalin, Refractory, and Healthy control groups using the analysis of variance (ANOVA). In the Antihistamine group, the CPT values at the baseline and week 2 were compared using the paired *t*-test. In the Pregabalin and Refractory groups, the CPT values between the baseline and week 2 and between weeks 2 and 4 were compared using the paired *t*-test.

*p < 0.05.

Unless otherwise indicated, p > 0.05.

### Results obtained at 250 Hz

The mean baseline CPT values for different groups, measured at 250 Hz on one forearm, were as follows: the Antihistamine group: 42.8; the Pregabalin group: 38.8; the Refractory group: 38.3; and the Healthy control group: 36.7. The mean CPT values recorded at 250 Hz on one forearm in patients from the Antihistamine, Pregabalin, and Refractory groups at weeks 2 and 4 are shown in Table 3.

**Table 3:** Baseline, week 2, and week 4 CPT values at 250 Hz.

CPT 250 Hz (Aδ-fibres)	Mean [SD] CPT values			
	Antihistamine group	Pregabalin group	Refractory group	Healthy control group
Baseline	42.8 [29.3]	38.8 [15.9]	38.3 [20.8]	36.7 [13.6]
Week 2	41.7 [19.6]	37.5 [17.9]	31.3 [12.5]	NA
Week 4	NA	40.3 [23.8]	48.7 [20.1]	NA

CPT, current perception threshold. NA, Not applicable.

At baseline, the CPT values were compared among the Antihistamine, Pregabalin, Refractory, and Healthy control groups using analysis of variance (ANOVA). In the Antihistamine group, the CPT values at the baseline and week 2 were compared using the paired *t*-test. In the Pregabalin and Refractory group, the CPT values between the baseline and week 2 and between weeks 2 and 4 were compared using the paired *t*-test.

Unless otherwise indicated, p > 0.05.

**Figure 2:**
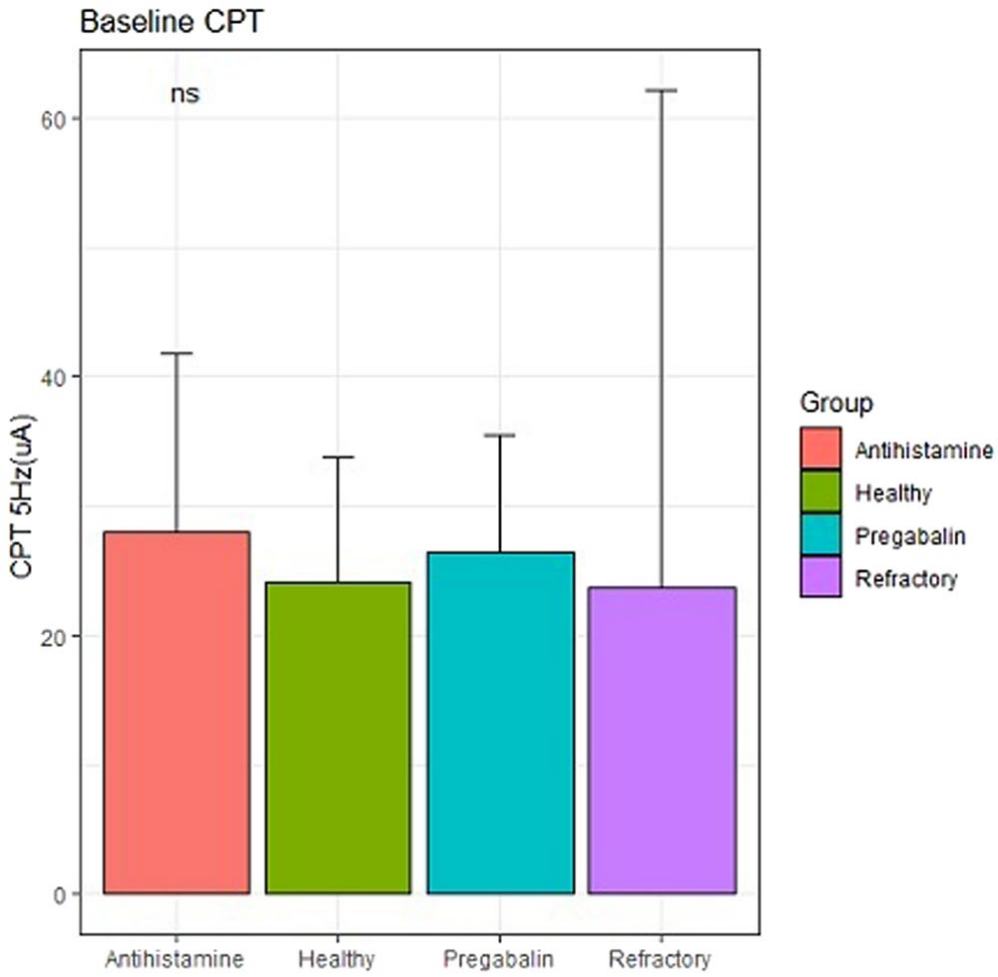
Baseline electric current perception threshold (CPT) values at 5 and 250 Hz on one forearm in patients with chronic pruritus of unknown origin (CPUO) from the Pregabalin, Antihistamine, and Refractory groups and in the healthy control individuals. CPT, electric current perception threshold; Lt, left; Rt, right.

**Figure 3:**
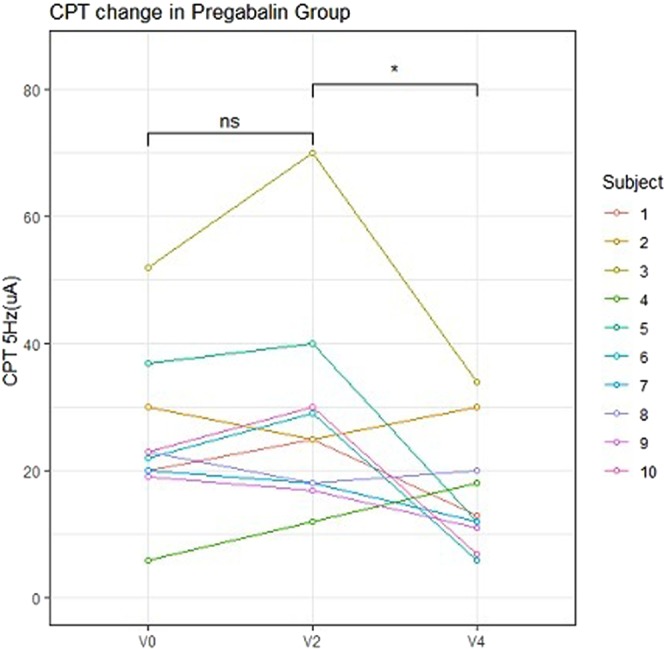
Changes in the CPT at 5 and 250 Hz with time in the Pregabalin group. CPT, electric current perception threshold; Lt, left; Rt, right; V0, baseline; V2, after treatment with two types of antihistamines; V4, after treatment with supplementary pregabalin; ns, not significant; *P < 0.05.

**Figure 4:**
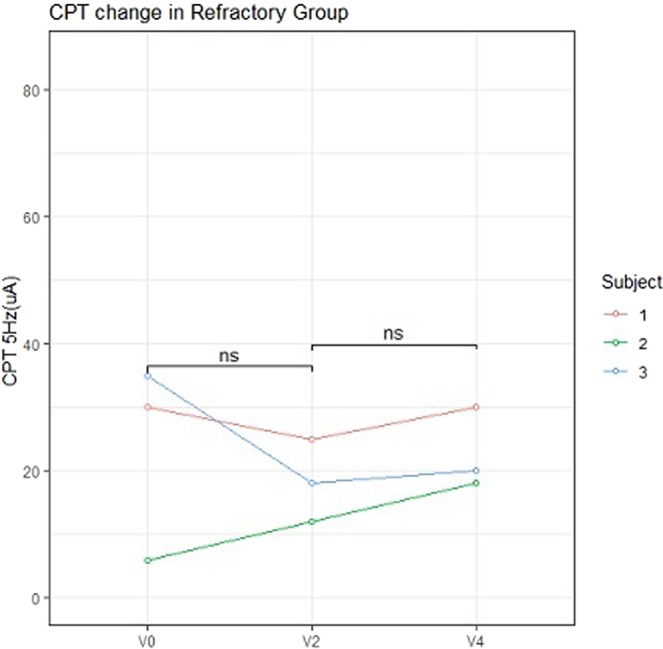
Changes in the CPT at 5 and 250 Hz with time in the Refractory group. CPT, electric current perception threshold; Lt, left; Rt, right; V0, baseline; V2, after treatment with two types of antihistamines; V4: after treatment with supplementary pregabalin; ns, not significant.

**Figure 5:**
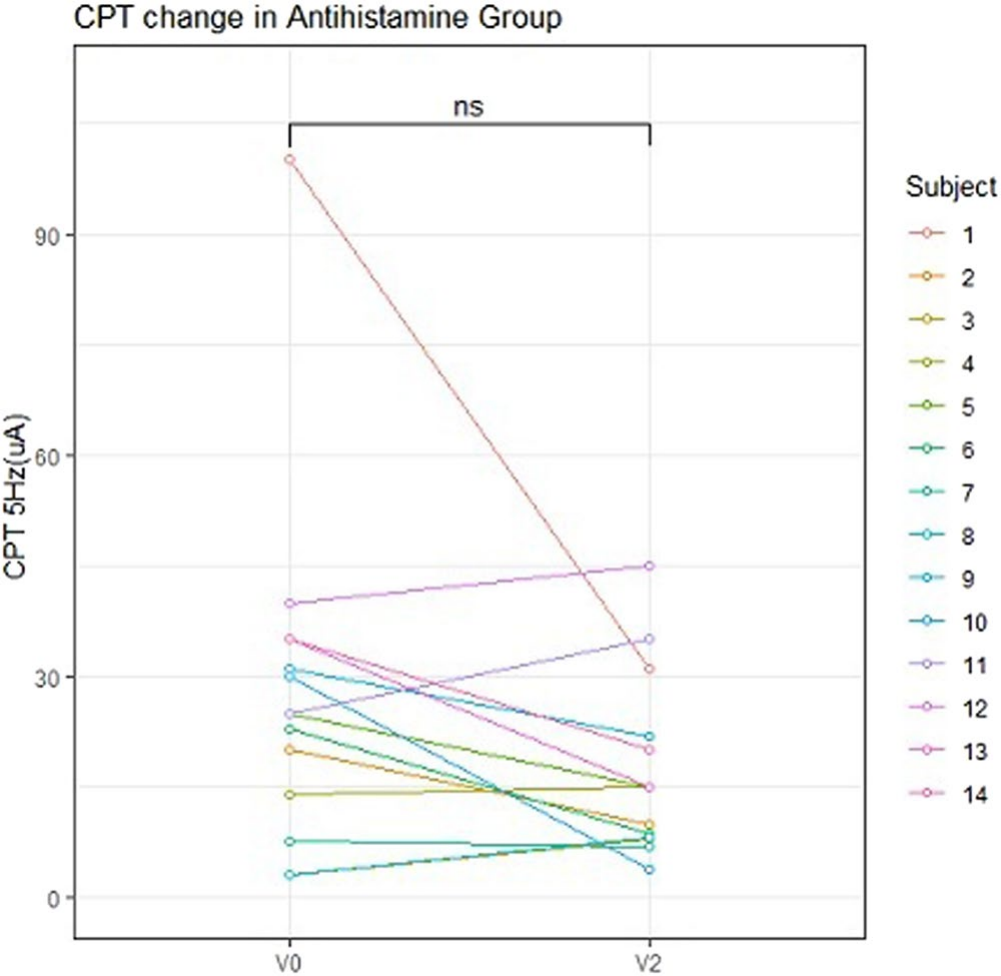
Changes in the CPT at 5 and 250 Hz with time in the Antihistamine group. CPT, electric current perception threshold; Lt, left; Rt, right; V0, baseline; V2, after treatment with two types of antihistamines; ns, not significant.

No significant differences were noted among the four study groups with respect to the baseline CPT values at either 5 or 250 Hz (Fig. 2). Seven of the 10 patients in the Pregabalin group reported an improvement in pruritus, although we detected no significant differences in the CPT values at 250 Hz between 2 and 4 weeks. In contrast, at 4 weeks (2 weeks after commencing pregabalin treatment), there was a significant decrease in the CPT values at 5 Hz on one forearm compared with those recorded at 2 weeks (Fig. 3). In the Refractory group, there were no significant differences in the CPT values at either 250 or 5 Hz between the baseline and 2 weeks and between 2 weeks and 4 weeks (Fig. 4). In the Antihistamine group, all participants reported improvements in pruritus; however, there were no significant differences in the CPT values at 250 and 5 Hz between the baseline and 2 weeks (Fig. 5). These results are summarised in Tables 2 and 3.

## Discussion

In this study, we found that the condition of 70% of patients with CPUO, who were refractory to second-generation antihistamine treatment, improved in response to the administration of pregabalin. Moreover, in these patients, there was a decrease in the CPT measured at 5 Hz, but not at 250 Hz. At baseline, we detected no significant differences in the 5- and 250-Hz CPT values among patients with CPUO and the healthy control individuals. Although CPUO was improved in some patients by second-generation antihistamines, we detected no significant changes in the CPT at 5 Hz. Similarly, patients with CPUO, whose condition was not improved by pregabalin (Refractory group), showed no significant changes in the CPT at 5 Hz.

CPUO has a considerable negative effect on the quality of life of individuals who suffer from this condition^1,9^. It is an itch-related disease of unknown aetiology, although there is no visible skin inflammation, in contrast to atopic dermatitis (AD)^10^. CPUO is known to be closely associated with ageing, and it has been suggested that patients with CPUO have a mild form of type 2 immunity, due to the loss of type 1 immunity^11,12^. In the present study, we also observed that the patients with CPUO showed no significant skin inflammation, despite presenting with severe pruritus. It has been reported that a subset of patients with CPUO is characterised by systemic type 2 inflammation, which is associated with reduced peripheral eosinophilia and elevated immunoglobulin E level^2^. Although there is considerably less evidence of skin inflammation in patients with CPUO, they are, nevertheless, more pruritic than patients with AD^10^, in whom the treatment for skin inflammation reduces their itching symptoms. In contrast, patients with CPUO often suffer from severe itching even under conditions of potent immunosuppression^10^. RNA sequencing analysis has indicated that at the molecular level, the skin of patients with CPUO is more comparable to that of patients with AD than to the skin of control individuals^10^. In addition, a gene set enrichment analysis revealed that numerous transcription factors could be used to distinguish CPUO and AD^10^. AD is characterised by widespread activation of the immune pathway in the skin, whereas CPUO is characterised by increased expression of the transcription factors associated with cell morphology and calcium in the skin^10^.

There are currently several devices that can be used to objectively measure pruritus, such as a vibration transducer attached to a bed leg, which can record whole-body movement at night, and movement-sensitive metre, which can measure limb movements at night^13–15^. Another method involves an *in vivo* model of pruritus induced by topical administration of histamine via needle injection or iontophoresis^16^. However, although potentially effective, these procedures tend to be inconvenient for use in an outpatient setting. In terms of physical stimulation, some studies have examined the efficacy of electrically induced itching to assess itching. In this regard, NEUROMETER CPT/C can be used to evaluate CPT^7^. Such sensory function monitoring is primarily used to diagnose diabetic neuropathy, but it can also be used in conjunction with the treatment of neurological diseases, including restless leg syndrome and mechanical neck disorder^17,18^. NEUROMETER CPT/C directly and differentially stimulates large- and small-diameter sensory nerve fibre, independent of factors such as skin thickness, temperature, and substances involved in the induction of pruritus^6,19^.

In animal studies, by examining the action potentials of rat dorsal root ganglion, C-fibres were activated only by transcutaneous sinewave at 5 Hz, not by 250 Hz and 2000 Hz^20^. Also, transcutaneous electrical stimulator (NEUROMETER CPT/C) at 5 Hz could provoke an itch sensation^21^. Two types of C-fibres are present in the human skin, mechano-responsive, and mechano-irresponsive C-fibres and the former did not mediate axon reflex flare^22,23^. Of note, electrically evoked itch was not accompanied by the axon flare reflex^21^. Therefore, the electrical stimulation at 5 HZ inducing itch would activate mechano-responsive C-fibres.

In the present study, similar to that carried out in patients with AD^7,24^, the baseline CPTs at 5 and 250 Hz, which respectively represented C-fibre and Aδ fibre, did not differ significantly between patients with CPUO and healthy control individuals. However, the CPT measurements obtained from the cheek at 5 and 250 Hz have not been found to significantly differ between patients with AD and healthy controls, but those obtained from the volar forearm were significantly lower in patients with AD than those obtained from the control individuals^24^. In patients with AD, the CPT appears to be affected by the itch state. Moreover, in patients with extrinsic AD, the CPT measurements at 5 Hz show a significant correlation with the VAS scores, and it has been found that pre-existing itching lowers the sensitivity to external stimuli^7^. In the itchy skin of patients with AD, the C-fibres have already been stimulated, and thus, may become desensitised in response to external stimuli^7^.

The U.S. Food and Drug Administration has approved pregabalin for the treatment of neuropathic pain, including diabetic peripheral neuropathy and postherpetic neuralgia. Pregabalin and gabapentin seem to be effective for chronic pruritus, and there is limited data comparing pregabalin and gabapentin^4^. However, pregabalin has some advantages over gabapentin, such as higher potency and faster absorption^25^. Besides, a preliminary study showed that pregabalin relieves pruritus in CPUO patients^26^. So, we chose pregabalin over gabapentin. In a sensitive state, characterised by severe inflammation, pregabalin suppresses the secretion of neuropeptides, including substance P and calcitonin gene-related peptides, from the spinal cord^27^. In patients with CPUO, the levels of neurotransmitters such as substance P may be chronically elevated, and consequently, the sensory nervous system, including C-fibres, can be chronically stimulated. By suppressing the release of certain neurotransmitters, pregabalin may resolve the stimulated state in these patients. Accordingly, they may experience a slight increase in their ability to perceive external stimuli, which is reflected in a reduced CPT at 5 Hz. The results of the present study thus indicate that pregabalin may relieve symptoms of pruritus by modulating the threshold of C-fibres. In addition, CPT could be used as an quantitative reference model, especially as a follow-up instrument, to monitor the improvement of pruritus in patients with CPUO.

It has previously been shown that neuronal Janus kinase 1 (JAK1) functions as a critical mediator of chronic itching, even in the absence of prominent skin inflammation^10^. Evidence showing that the disruption of neuronal JAK1 signalling in response to treatment with tofacitinib limits non-inflammatory itching suggests that inhibition of JAK1 may represent a novel neuromodulatory approach to target itching^10^. The interleukin-6 (IL-6)/JAK2/signal transducer and activator of transcription 3 (STAT3) pathways are implicated in the development of neuropathic pain and synaptic plasticity^28,29^. In this regard, the administration of pregabalin has been shown to significantly lower the peripheral neuropathic pain induced by paclitaxel by suppressing the IL-6/JAK2/STAT3 pathway in nerves, which is probably associated with a reduction in the IL-6 content^30^. Thus, it is conceivable that pregabalin reduces itching.

A limitation of the present study is that several participants had to be excluded from the analysis because they did not attend the required regular follow-ups. Furthermore, the number of participants was small, especially in the Pregabalin (n = 7) and Refractory (n = 3) groups. Therefore, further, more extensive studies are needed to validate this result. Besides, it can be argued that instead of administering 150 mg/day pregabalin for only 2 weeks, increasing the dose or prolonging the duration of treatment might have resulted in a more pronounced response in patients with CPUO. Moreover, we were unable to monitor the long-term changes in CPT or pruritus symptoms after the cessation of pregabalin treatment. The predominance of males among the patients with CPUO was not ideal for minimising any potential bias between patients with CPUO and healthy controls. Finally, CPT test requires the detailed patient’s provoked itch responses, so the patients must be able to communicate with testers. Therefore, CPT test could not be applied to patients with mental illness or unable to properly communicating with testers.

In conclusion, there was no difference in the baseline CPT between patients with CPUO and healthy control. Pregabalin improved pruritus by lowering the threshold of C-fibre, as CPT, a surrogate marker of the C-fibre threshold, decreased with the improvement of pruritus by pregabalin. This study is, to the best of our knowledge, the first to show that the measurement of CPT can be used as an objective method to verify the improvement in CPUO in response to the administration of pregabalin. However, the appropriate dose and/or treatment duration with pregabalin to maximise its effects remain to be determined. Further studies are also needed to establish a subset of patients that shows the best results.

## Data Availability

The datasets generated during and/or analysed during the current study are available from the corresponding author on reasonable request.

## References

[1] Yosipovitch G, Bernhard JD (2013). Clinical practice. Chronic pruritus. N. Engl. J. Med..

[2] Xu AZ, Tripathi SV, Kau AL, Schaffer A, Kim BS (2016). Immune dysregulation underlies a subset of patients with chronic idiopathic pruritus. Journal of the American Academy of Dermatology.

[3] Kim BS, Berger TG, Yosipovitch G (2019). Chronic pruritus of unknown origin (CPUO): Uniform nomenclature and diagnosis as a pathway to standardized understanding and treatment. Journal of the American Academy of Dermatology.

[4] Matsuda KM, Sharma D, Schonfeld AR, Kwatra SG (2016). Gabapentin and pregabalin for the treatment of chronic pruritus. J. Am. Acad. Dermatol..

[5] Cevikbas F, Steinhoff M, Ikoma A (2011). Role of spinal neurotransmitter receptors in itch: new insights into therapies and drug development. CNS Neurosci. Ther..

[6] Katims JJ, Naviasky EH, Ng LK, Rendell M, Bleecker ML (1986). New screening device for assessment of peripheral neuropathy. J. Occup. Med..

[7] Mori T (2010). Comparison of skin barrier function and sensory nerve electric current perception threshold between IgE-high extrinsic and IgE-normal intrinsic types of atopic dermatitis. Br. J. Dermatol..

[8] R Development Core Team. R: a language and environment for statistical computing (2018).

[9] Matterne U (2011). Prevalence, correlates and characteristics of chronic pruritus: a population-based cross-sectional study. Acta Derm. Venereol..

[10] Oetjen, L. K. *et al*. Sensory Neurons Co-opt Classical Immune Signaling Pathways to Mediate Chronic Itch. Cell 171, 217-228.e13 (2017). 10.1016/j.cell.2017.08.006 PMC5658016 28890086

[11] Reich A, Stander S, Szepietowski JC (2011). Pruritus in the elderly. Clin. Dermatol..

[12] Berger TG, Steinhoff M (2011). Pruritus in elderly patients–eruptions of senescence. Semin. Cutan. Med. Surg..

[13] Ebata T, Iwasaki S, Kamide R, Niimura M (2001). Use of a wrist activity monitor for the measurement of nocturnal scratching in patients with atopic dermatitis. Br. J. Dermatol..

[14] Felix R, Shuster S (1975). A new method for the measurement of itch and the response to treatment. Br. J. Dermatol..

[15] Wahlgren CF (1995). Measurement of itch. Semin. Dermatol..

[16] Heyer G, Hornstein OP, Handwerker HO (1989). Skin reactions and itch sensation induced by epicutaneous histamine application in atopic dermatitis and controls. J. Invest. Dermatol..

[17] Uddin Z, MacDermid JC, Galea V, Gross AR, Pierrynowski MR (2014). The current perception threshold test differentiates categories of mechanical neck disorder. J. Orthop. Sports Phys. Ther..

[18] Cho YW (2017). Quantitative sensory test for primary restless legs syndrome/Willis-Ekbom disease using the current perception threshold test. Sleep Med..

[19] Tay B, Wallace MS, Irving G (1997). Quantitative assessment of differential sensory blockade after lumbar epidural lidocaine. Anesth. Analg..

[20] Koga K (2005). Selective activation of primary afferent fibers evaluated by sine-wave electrical stimulation. Mol. Pain.

[21] Ozawa M (2006). Neuroselective transcutaneous electric stimulation reveals body area-specific differences in itch perception. J. Am. Acad. Dermatol..

[22] Schmelz M (2000). Which nerve fibers mediate the axon reflex flare in human skin?. Neuroreport.

[23] Schmidt R (1995). Novel classes of responsive and unresponsive C nociceptors in human skin. J. Neurosci..

[24] Kobayashi H, Kikuchi K, Tsubono Y, Tagami H (2003). Measurement of electrical current perception threshold of sensory nerves for pruritus in atopic dermatitis patients and normal individuals with various degrees of mild damage to the stratum corneum. Dermatology.

[25] Bockbrader HN (2010). A comparison of the pharmacokinetics and pharmacodynamics of pregabalin and gabapentin. Clin. Pharmacokinet..

[26] Park J-M (2012). Efficacy and safety of pregabalin for the treatment of chronic pruritus in Korea. The Journal of dermatology.

[27] Fehrenbacher JC, Taylor CP, Vasko MR (2003). Pregabalin and gabapentin reduce release of substance P and CGRP from rat spinal tissues only after inflammation or activation of protein kinase C. Pain.

[28] Nicolas CS (2013). The role of JAK-STAT signaling within the CNS. JAK-STAT.

[29] Xue Z-J (2014). STAT3 inhibitor WP1066 as a novel therapeutic agent for bCCI neuropathic pain rats. Brain Res..

[30] Al-Massri KF, Ahmed LA, El-Abhar HS (2018). Pregabalin and lacosamide ameliorate paclitaxel-induced peripheral neuropathy via inhibition of JAK/STAT signaling pathway and Notch-1 receptor. Neurochem. Int..

